# Analysis of risk factors for postoperative dysphagia after C1-2 fusion

**DOI:** 10.3389/fsurg.2022.977500

**Published:** 2022-10-13

**Authors:** Dong Sun, Jianhui Mou, Zhaolin Wang, Peng Liu

**Affiliations:** Department of Orthopaedics, China-Japan Union Hospital of Jilin University, Changchun, Jilin, China

**Keywords:** postoperative dysphagia, C1-2 fusion surgery, oropharyngeal space, cervical sagittal alignment, risk factors

## Abstract

**Objective:**

This study aimed to analyze the risk factors for dysphagia after **C1-2** fusion in patients with C1-2 junction diseases.

**Summary of the background data:**

Dysphagia is a common postoperative complication of posterior C1-2 junction surgery. The incidence is 9.5% to 26.3%. However, the etiopathogenisis of postoperative dysphagia remains controversial.

**Methods:**

This retrospective study included patients who underwent **C1-2** fusion from January 2016 to January 2020. The patients were divided into dysphagia group and control group in accordance with the Bazaz R dysphagia scoring system. The patients' age, gender, BMI(body mass index), cause of disease, and changes in the C01cobb, C02cobb, C12cobb, C27cobb, dC02cobb, dC01cobb, dC12cobb, d C27cobb angles before and after operation, were recorded. The parameters and changes were compared to analyze the risk factors for dysphagia after **C1-2** fusion.

**Results:**

65 cases (15, with dysphagia; 50, without dysphagia) were included. The incidence of postoperative dysphagia was 23%. The differences in age, gender ratio, and BMI between the two groups were not significant (*P* > 0.05). The differences among postoperative C12 (29.8° ± 11.24° vs. 20.46° ± 13.39°), postoperative C27cobb (10.56° ± 8.53° vs. 20.21° ± 13.21°), and dC12cobb (9.49° ± 5.16° vs. 1.07° ± 12.44°) between the two groups were significant (*P* < 0.05). Multiple logistic regression analyses revealed that dC12cobb > 5° was a significant independent risk factor for postoperative dysphagia, And preoperative C27cobb was a preventive factor of postoperative dysphagia.

**Conclusions:**

Dysphagia after the **C1-2** fusion was common. dC02cobb and dC12cobb were the significant independent risk factors for postoperative dysphagia. Preoperative c27cobb was a preventive factor of dysphagia.

## Highlights

When making an operation scheme, the surgeon should pay attention to the change in the alignment of C1-2 junction area, in order to avoid alignment over changes during operation, and maintain C02cobb and C12cobb in the normal range to prevent postoperative dysphagia.

Multiple logistic regression analyses revealed that dC02cobb and dC12cobb were the significant independent risk factors for postoperative dysphagia.

Preoperative c27cobb was a preventive factor of dysphagia.

## Introduction

Postoperative dysphagia, as a common complication of the C12 fusion surgery, affects the patients' daily life and endangers their lives (1, 2). According to the literature, the incidence rate of postoperative dysphagia ranges from 9.5% to 26.3% (3–6). However, the etiopathogenisis of postoperative dysphagia, including the decrease in postoperative C02cobb and oropharyngeal space, changes in the preoperative and postoperative C02cobb, and the change in postoperative C27cobb, remain controversial (7–9).

In this study, the clinical data of the patients who received posterior fixation and fusion in the C12 region due to the lesions in the C1-2 region were analyzed retrospectively. In accordance with the occurrence of postoperative dysphagia and changes in cervical sagittal alignment, a case–control study was carried out to clarify the relationship between postoperative dysphagia and cervical sagittal alignment in the craniocervical junction area and explore the risk factors for postoperative dysphagia.

## Materials and methods

### Patients

The data of 65 consecutive patients with posterior cervical fusion from January 2016 to January 2020 were analyzed retrospectively. The inclusion criteria were patients (1) with congenital, traumatic, or neoplastic craniocervical lesions; (2) with posterior C12 fusion; (3) with complete preoperative and postoperative imaging data and at least 3 months of follow-up information; and (4) older than 18 years. The exclusion criteria were patients (1) with previous occipitocervical operation, (2) with incomplete imaging data and were lost to follow-up, (3) with dysphagia and/or dyspnea before operation, and (4) who did not undergo operation due to contraindications. In accordance with the inclusion and the exclusion criteria, 65 patients with an average age of 45 years (18–68 years old) were included in this study.

In the Bazaz R dysphagia evaluation, patients without dysphagia were placed into the asymptomatic (control) group, and those who had mild, moderate, and severe dysphagia were placed into the dysphagia group (10). Patients with no dysphagia were graded as “None”. Only patients with rare dysphagia episodes were graded as “mild”. “Moderate” dysphagia is defined as the occasional swallowing of very special foods (such as bread or steak). “Severe” dysphagia was defined as frequent difficulty with most foods. “None” and “Mild” patients without obvious dysphagia were included in the asymptomatic group. “Moderate” and “Severe” patients were included in the symptom group. 15 patients were included in the dysphagia group, and 50 patients were included in the asymptomatic group. Among the 65 patients, 32 were traumatic C1-2 lesions, 25 were degenerative, and 8 were tumors. Patients' dysphagia during postoperative hospitalization was evaluated by doctors who knew the bazaz R dysphagia scoring system. After discharge, the patient's telephone follow-up was carried out by the doctor who evaluated the hospitalization. Evaluate the degree of dysphagia and recovery time after discharge. All operations were performed by senior surgeons with more than 10 years of clinical experience in our center. This study was approved by the Ethics Committee of Jilin University School of Clinical Medicine, and written informed consent forms were obtained from all participants.

### Surgical technique

All patients were treated with C1 lateral mass or posterior arch screw and C2 pedicle screw fixation (11, 12). After intubation in general anesthesia, the patients were placed in the prone, headrest-fixed head, neck neutral, or mild extension position. The central point of the inferior articular process of the C1 lateral mass or the corresponding central point of the upper and the lower edge of the C1 posterior arch was selected as the insertion point. On the basis of the preoperative imaging measurement, 15° head inclination and 5° to 10° medial inclination were adjusted. The insertion point of the C2 screw was located at the projection point of the articular process of the internal and the external edges of the pedicle. 20° head inclination and 30° medial inclination were adjusted. After the implantion of the screw, the atlantoaxial joint was reduced. The posterior titanium rod was implanted to complete the reduction by segmental compression and backward pulling of C1 vertebral body. The iliac bone was used for posterior atlantoaxial fusion, drainage, and suture layer by layer. Tracheal intubation was removed When the patient woke up.

### Postoperative management

After lying in bed for 24 h, the patients carried out activities by using a neck bracket. And the instrument position and atlantoaxial reduction were examined using an x-ray film. The wound drainage was less than 30 ml after 48 h. The neck bracket was fixed 4–6 weeks later. And bone fusion was confirmed through reexamination three months later.

### Clinical and imaging data

**Figure 1:**
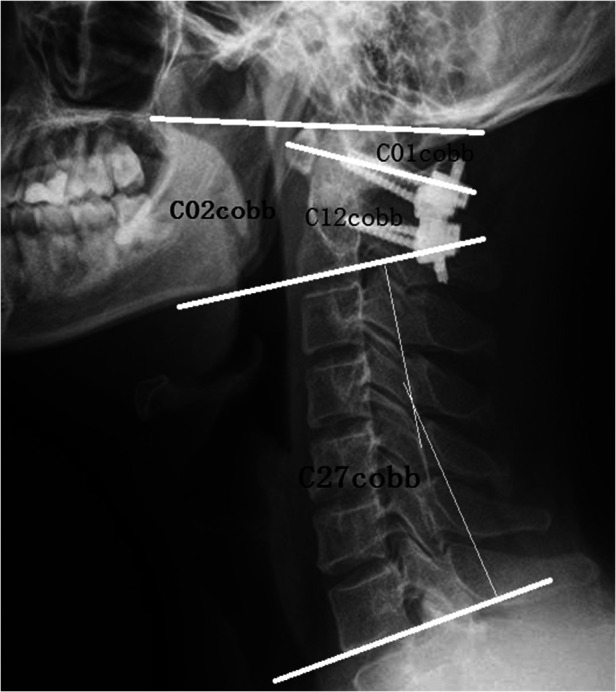
The measurement method of cervical spinal alignment.

The general clinical data included age, gender, and BMI. The following imaging data were measured in accordance with the AP(Anteroposterior) and lateral radiographs of the cervical spine before and after operation: (1) C02cobb angle (the angle between the McGregor lines and the C2 vertebral body's lower endplate lines), (2) C01cobb (the angle between the McGregor lines and the C1 anterior and posterior arches' midpoint lines), (3) C12cobb (the angle between the lines of C1 anterior and posterior arches' midpoint and the C2 vertebral body's lower endplate lines), (4) C27cobb (the angle between the C2 and the C7 vertebral body's lower endplate lines), and (5) the changes in the angles of C02cobb, C01cobb, C12cobb, and C27cobb before and after operation (dC02cobb, dC01cobb, dC12cobb, and dC27cobb, respectively) (Figure 1). The post-processing tasks were performed by two well-trained reviewers, a research assistant (reviewer A) with 3 years of experience and a radiology technologist (reviewer B) with 1 year of experience with the equipment. To assess intraobserver repeatability, the variables for each group were measured twice by each reviewer at intervals of 2 weeks. The measurement results were considered consistent if the ICC value was greater than 0.80, Kappa coefficient was used to evaluate the consistency of variables between the two groups, which was greater than 0.80. The ICC coefficients were 0.83, 0.90, 0.84 and 0.80 for C02cobb, C01cobb, C12cobb, and C27cobb respectively.

### Statistical analysis

All data were analyzed using the SPSS 22.0 (SPSS Inc., Chicago, IL, USA). The patients were divided into the dysphagia and the asymptomatic groups in accordance with the presence or absence of dysphagia. Descriptions involving normal distribution and continuous measures were presented as mean ± standard deviation. The Student's *t*-test was used to analyze the differences in the general and the image data between groups. The classification variables (gender) were examined using the Fisher's exact test (bilateral). After the elimination of gender interference, the logistic regression analysis confirmed the independent risk factors for postoperative dysphagia. Bivariate linear regression analysis was used to evaluate the associations between different variables with the presence of dysphagia and then a multivariate analysis adjusting with confounding variables. For the positive risk factors, the ROC curve was drawn to determine the cutoff value of dysphagia and the corresponding sensitivity and specificity. All reported *P*-values were two-tailed, and the level of statistical significance was *P* < 0.05.

## Results

### General information

A total of 65 patients were included, of which 15 were included in the dysphagia group (10 males and 5 females), and 50 were included in the asymptomatic group (33 males and 17 females). The average age and average BMI of the patients in the dysphagia group were 52 years (36–67 years) and 24 (18–29), respectively, whereas those in the asymptomatic group were 48.14 years (18–68 years) and 23 (17–28), respectively. The incidence of dysphagia was 23%. The demographic data of both groups were shown in Table 1. The differences between the two groups in terms of age and male-to-female ratio were not significant (Table 1).

**Table 1:** Comparison of general information in dysphagia and asymptomatic groups.

Group	Dysphagia	Asymptomatic	*P*
Age (years)	52.6 ± 9.08	48.3 ± 13.9	>0.05
Gender (Male/Female)	16/8	40/23	>0.05
BMI	24.13 ± 3.35	23.89 ± 2.58	>0.05

### Analysis of the imaging data

**Figure 2:**
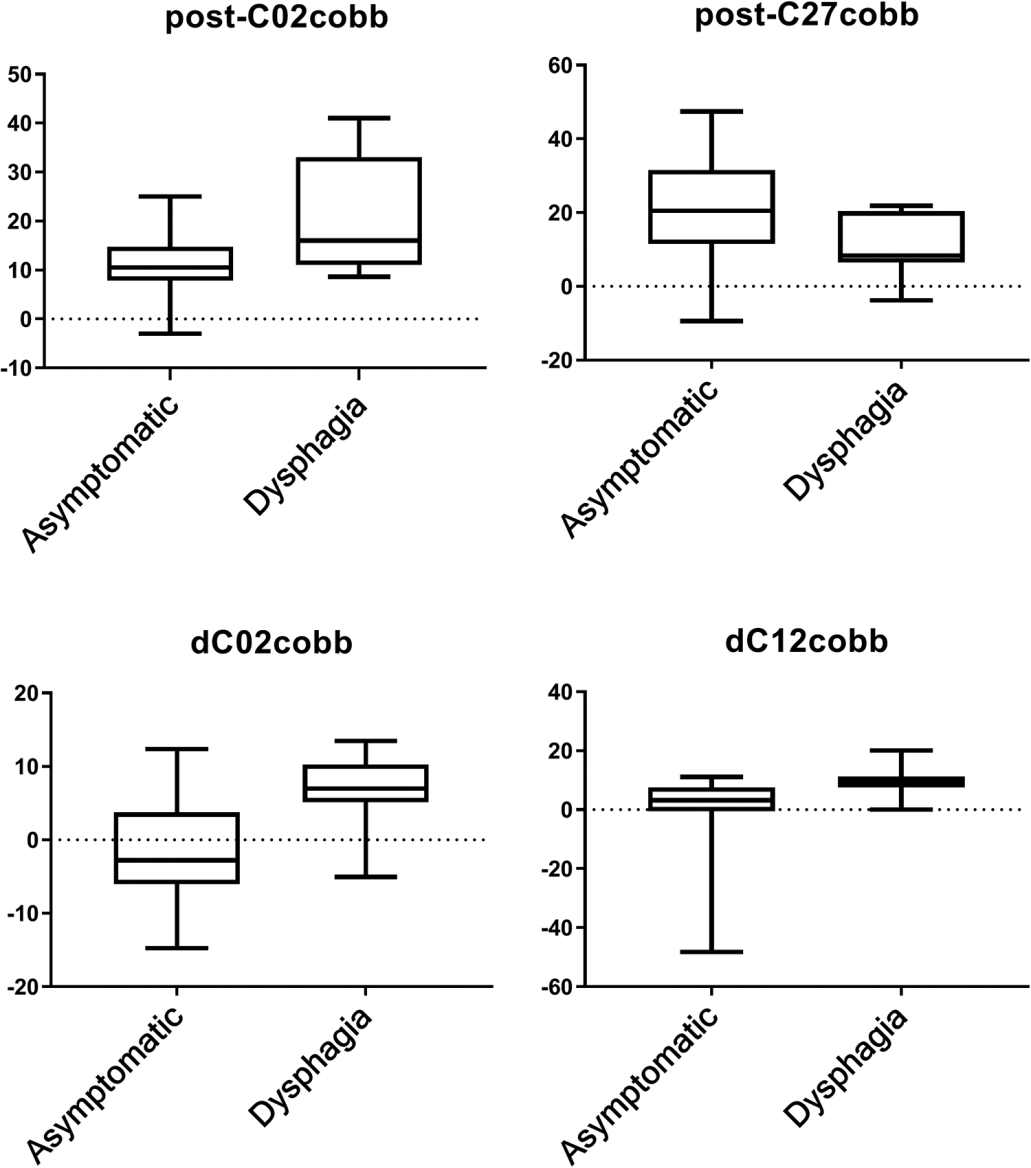
Comparison of cervical alignment between dysphagia and asymptomatic groups.

**Figure 3:**
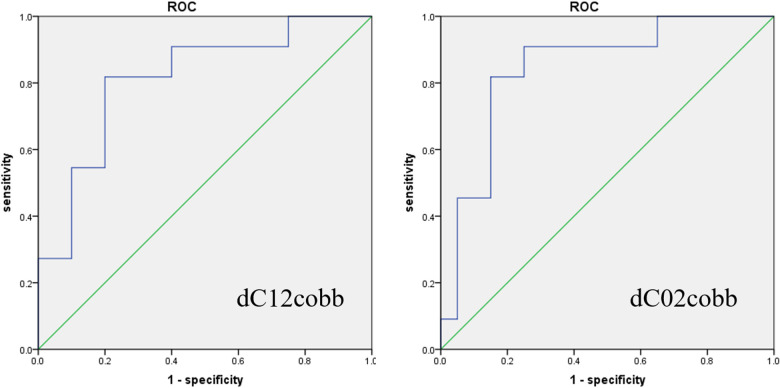
Receiver operating characteristic (ROC) curve. The area under the ROC in terms of dC02cobb and dC12cobb was 0.845,0.818.

The results of the Student's *t*-test showed significant differences (*P* < 0.05) between the two groups in terms of postoperative C12(29.8° ± 11.24°vs. 20.46° ± 13.39°), and C27cobb (10.56° ± 8.53° vs. 20.21° ± 13.21°) (Tables 2, 3, Figure 2). There was no significant differences in terms of C02 (20.34° ± 11.29° vs. 16.92° ± 6.89°) between two groups. The logistic regression analysis showed that dC12cobb was the independent risk factors for postoperative dysphagia, and their OR values were 1.311(*P* = 0.034). DC02cobb was the independent risk factors for postoperative dysphagia, and their OR values were 1.593(*P* = 0.009). Preoperative C27cobb was the preventive factor of postoperative dysphagia, and its OR(odds ratio) value was 0.883 (*P* = 0.04) (Table 4). The ROC(receiver operating curve) curve of the parameters dC12cobb was drawn, as shown in the Figure 3. The AUC, cutoff, sensitivity, and specificity values of dC12cobb were 0.818, 7.5°, 0.818, and 0.80, respectively.

**Table 2:** Comparison of cervical alignment between dysphagia and asymptomatic groups (°).

Group	PreC02	PreC01	PreC12	PreC27	PostC02	PostC01	PostC12	PostC27
Dysphagia	13.62 ± 11.79	−6.39 ± 5.83	20.32 ± 11.16	12.4 ± 11.41	20.34 ± 11.29	−9.49 ± 6.58	29.80 ± 11.24	10.56 ± 8.54
Asymptomatic	12.80 ± 10.24	−6.67 ± 8.86	19.39 ± 6.75	20.78 ± 11.25	16.92 ± 6.89	−10.52 ± 7.89	20.46 ± 13.39	20.21 ± 13.21
*P*	0.841	0.925	0.775	0.058	0.07	0.715	0.04	0.038

**Table 3:** Comparison of pro- and post- cervical alignment between dysphagia and asymptomatic groups (°).

Group	Post-PreC02 (dC02)	Post-PreC01 (dC01)	Post-PreC12 (dC12)	Post-PreC27 (dC27)
Dysphagia	3.72 ± 4.85	−3.09 ± 4.12	9.49 ± 5.16	1.85 ± 4.64
Asymptomatic	−1.89 ± 7.21	−3.85 ± 10.84	1.07 ± 12.44	−0.57 ± 11.76
*P*	0.06	0.845	0.041	0.734

**Table 4:** Logistic regression analysis for the influence of predictors on postoperative dysphagia.

Predictors	B	SE	WALS	OR (95% CI)	*P*
Post-PreC01 (dC01)	0.271	0.128	4.561	1.311 (1.021–1.684)	0.034
Post-PreC02 (dC02)	0.466	0.178	6.860	1.593 (1.124–2.258)	0.009
Pre- C27cobb	−0.124	0.060	4.220	0.883 (0.785–0.994)	0.040

## Discussion

The C12 fusion operation is difficult due to the complex anatomy and postoperative complications, including vertebral artery injury, cerebrospinal fluid leakage, surgical infection, postoperative dysphagia, nerve injury, instrument complications, and bone fusion failure (13). Postoperative dysphagia is a common complication of posterior craniocervical junction surgery. According to the previous literature, the incidence is 9.5% to 26.3% (3–6). The incidence of dysphagia is 23% in this study. However, most patients are relieved within few months after surgery. Makoto (14) has reported that patients with severe dysphagia couldn't be relieved because of the abnormal alignment of the upper cervical vertebra after surgery and should undergo revision surgery to improve postoperative dysphagia (14). In previous studies, most patients underwent occipitocervical fusion. The craniocervical junction sequence was fixed because of occipitocervical fusion, and it was difficult to relieve dysphagia, Mazhar Iqbal (15) proved that patients with dysphagia after occipitocervical fusion did not relieve and underwent reoperation. However, there were few studies on dysphagia after C12 fusion. In this study, patients were treated with C12 fusion, and the patients were relieved within 6 months after operation, which might be due to the compensation mechanism of C01 segment after C12 fusion.

**Figure 4:**
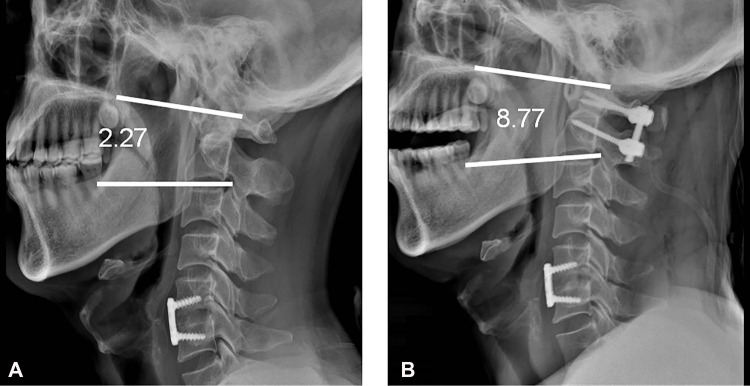
A case of postoperative dysphagia. (**A**) The preoperative C02cobb was 2.27°. (**B**) The postoperative C02cobb was 8.77°. dC02cobb was 6.5°.

Although the incidence of postoperative dysphagia is high, the pathogenesis of dysphagia is still controversial. Izeki (16) indicated a significant correlation between the angle of C02cobb and the space of the oropharynx after upper cervical fusion. The main reason for postoperative dysphagia was the decrease in the angle of C02cobb caused by the fusion of C02 in the flexion. Midori (17) found that the angle of C02 in dysphagia group decreased significantly in patients with halo-vest fixation, but the angle of C01 and C12 could not be analyzed. Masato (18) proved that the alignment of the upper cervical vertebra determined the change in oropharyngeal space by the variation in the oropharyngeal space of volunteers in different postures. However, whether a definite correlation exists between the decrease in oropharyngeal space and the occurrence of dysphagia is not clear, and whether the decrease in the oropharyngeal space is the mechanism leading to dysphagia is still uncertain. In oropharyngeal space measurement, the head posture and the postoperative swelling of tissues around the pharyngeal cavity lead to various errors. Yang (19) analyzed volunteers and patients after posterior upper cervical fusion and believed that the angle of C02cobb was the key factor for dysphagia and that the angle of dC02cobb less than −5° is critical. Morizane et al. (20). proposed a novel parameter: occipital and external acoustic meatus to axis angle (O-EAa). They aimed to address the shortcomings of O-C2cobb, which showed a wide deviation and couldn't reflect the translational changes in the cranium in relation to C2 (21, 22). The relationship between O-C2cobb/O-EAa and postoperative dysphagia has been discussed in several studies (20–23). Wang et al. (24). demonstrated that PIA had a similar predictive effect as O-EAa and could be used as a predictor for postoperative dysphagia in patients undergoing OCF(Occipitocervical fusion book). Tian et al. (25). analyzed the situation of dysphagia after anterior and posterior cervical surgery and believed that the main cause of dysphagia was related to the change in C27cobb. In the present study, the dysphagia of patients after upper cervical C12 fusion in our center was analyzed. It was shown that the angle of C27cobb in the dysphagia group was significantly smaller than that in the asymptomatic group (*P* = 0.038), whereas the angle of C12cobb in the dysphagia group is significantly larger than that in the asymptomatic group (*P* < 0.05). The angle of C12cobb might be enlarged by hyperextension and C12 fusion, which evidently changed the cervical vertebra alignment postoperation compared with that preoperation. This change might be the reason of dysphagia after the C12 fusion (Figure 4).

Yang (19) believed that the dO-C2A should be a key factor in the development of postoperative dysphagia after OCF. OTA et al. (26). analyzed the relationship between the C02cobb and the change in oropharyngeal space in volunteers and found that the decrease in C02cobb by 10° reduced the oropharyngeal space by 40%. Misawa et al. (27). reported that a patient with Klippel Feil syndrome had developed pharyngeal discomfort, dysphagia, and dysphagia after the internal fixation of the posterior C12 screw. The lateral x-ray showed that the occipitocervical angle (the angle between the McGregor line and the tangent line of the lower edge of the axis) decreased from 37° to 27°, and the head position was fixed in the flexion position. When the occipitocervical angle increaseed to 43°, dysphagia was improved. The authors speculated that the occipitocervical angle was an important factor in dysphagia. Izeki et al. (16). suggested that even a small change in the C02 angle would cause a significant change in the oropharyngeal space. However, in the occipitocervical fusion operation, if the anterior approach was not released, the main sagittal sequence changes would occur in C01, and the angle of C12 would be relatively fixed. Whether the dysphagia is due to the C01 hyperkyphosis has not been analyzed. In the present study, the changes in the cervical alignment of the dysphagia and the asymptomatic groups before and after operation were compared. Significant differences (*P* < 0.05) were shown in dC12cob (9.49° ± 5.16° vs. 1.07° ± 12.44°). However, unlike the results of previous studies, the angles of C12 in the dysphagia group increased significantly after operation in this study. The postoperative dysphagia might arised from that the changes in the alignment of the upper cervical vertebra exceeded the cutoff value, regardless of whether it was flexion or hyperextension state fusion. If the alignment change in the upper cervical vertebra exceeded the certain cutoff value, the anatomical state of the craniocervical junction area would be destroyed, thereby leading to dysphagia.

Logistic regression analysis excluding the classification variables (gender)suggested that dC12cobb was the independent risk factor for postoperative dysphagia (OR values = 1.311, Range:1.021–1.684, *P* = 0.034). DC02cobb was the independent risk factors for postoperative dysphagia, and their OR values were 1.593(Range: 1.124–2.258, *P* = 0.009). The preoperative C27cobb lordosis was the preventive factor of postoperative dysphagia (OR value = 0.883, Range: 0.785–0.994, *P* = 0.04). The ROC curves were drawn using the dC12cobb data with cutoff value of 7.5°. This result was consistent with the critical value proposed by Yang (19). When the change in the angle of C12cobb postoperation was more than 7.5°, the sensitivity and specificity of dysphagia after operation were 0.818 and 0.80, respectively. This result further confirmed that the excessive change the alignment in cervical vertebra alignment (e.g., C12cobb) after operation, compared with that before operation, would lead to postoperative dysphagia. In order to prevent postoperative dysphagia, the surgeon should pay special attention to the changes in the alignment of the upper cervical vertebra before making the preoperative plan and implementing the operation.

In the aspect of swallowing function recovery, Tomohiro (28) reported that the hypoglossal nerve palsy, a cause of severe dysphagia along with the orthopharyngeal stenosis due to occipitocervical kyphosis, could improve swallowing function after correction of kyphosis. Kimo et al. (29). concluded that neurogenic dysphagia associated with vagal nerve dysfunction. Mazhar Iqbal (15) emphasized that in the case of C1/C2 instability, it was preferable to perform C1/C2 fusion rather than OCF. In this study, all patients had C12 instability and underwent C12 fusion. Dysphagia was significantly relieved within 6 months after operation. It was further proved that dysphagia caused by postoperative sagittal sequence change could be compensated by sagittal sequence change of craniocervical junction area (such as C01 angle change).

The limitations of this study are as follows: (1) retrospective study, (2) small sample size, (3) failure to make extensive changes in the oropharyngeal space by routine CT during follow-up, and (4) failure to make remarkable changes in the oropharyngeal space by laryngoscopy in most patients. In the follow-up, a multicenter prospective study with a large sample size should be carried out to determine the causes of dysphagia after posterior upper cervical fusion.

## Conclusion

Postoperative dysphagia is a common complication of C12 fusion surgery. The changes in the preoperative and postoperative angles of C02cobb and C12cobb may be the risk factors for postoperative dysphagia. The excellent preoperative curvature of the lower cervical spine C27cobb can prevent postoperative dysphagia. When creating an operation plan, the surgeon should pay attention to the change in the alignment of the craniocervical junction area, in order to avoid alignment over changes during operation, and maintain C02cobb and C12cobb in the normal range to prevent postoperative dysphagia.

## Data Availability

The original contributions presented in the study are included in the article/Supplementary Material, further inquiries can be directed to the corresponding author/s.
